# Genome-Wide Association Study Using Whole-Genome Sequencing Identifies a Genomic Region on Chromosome 6 Associated With Comb Traits in Nandan-Yao Chicken

**DOI:** 10.3389/fgene.2021.682501

**Published:** 2021-08-02

**Authors:** Zhuliang Yang, Leqin Zou, Tiantian Sun, Wenwen Xu, Linghu Zeng, Yinhai Jia, Jianping Jiang, Jixian Deng, Xiurong Yang

**Affiliations:** ^1^College of Animal Science and Technology, Guangxi University, Nanning, China; ^2^Guangxi Institute of Animal Science, Nanning, China; ^3^Guangxi Botanical Garden of Medicinal Plants, Nanning, China

**Keywords:** genome-wide association study, Nandan-Yao chicken, whole-genome sequencing, comb traits, SNPs, INDELs

## Abstract

Comb traits have potential economic value in the breeding of indigenous chickens in China. Identifying and understanding relevant molecular markers for comb traits can be beneficial for genetic improvement. The purpose of this study was to utilize genome-wide association studies (GWAS) to detect promising loci and candidate genes related to comb traits, namely, comb thickness (CT), comb weight (CW), comb height, comb length (CL), and comb area. Genome-wide single-nucleotide polymorphisms (SNPs) and small insertions/deletions (INDELs) in 300 Nandan-Yao chickens were detected using whole-genome sequencing. In total, we identified 134 SNPs and 25 INDELs that were strongly associated with the five comb traits. A remarkable region spanning from 29.6 to 31.4 Mb on chromosome 6 was found to be significantly associated with comb traits in both SNP- and INDEL-based GWAS. In this region, two lead SNPs (6:30,354,876 for CW and CT and 6:30,264,318 for CL) and one lead INDEL (a deletion from 30,376,404 to 30,376,405 bp for CL and CT) were identified. Additionally, two genes were identified as potential candidates for comb development. The nearby gene fibroblast growth factor receptor 2 (*FGFR2*)—associated with epithelial cell migration and proliferation—and the gene cytochrome b5 reductase 2 (*CYB5R2*)—identified on chromosome 5 from INDEL-based GWAS—are significantly correlated with collagen maturation. The findings of this study could provide promising genes and biomarkers to accelerate genetic improvement of comb development based on molecular marker-assisted breeding in Nandan-Yao chickens.

## Introduction

The Nandan-Yao chicken, with a single comb, is a typical indigenous breed in Guangxi Zhuang Autonomous Region (Yang et al., 2020). To meet the particular demand of local customers, chickens with a prominent ornament are preferred breeding targets for the local poultry industry.

The comb is a sexual ornament in chickens, and its morphological characteristics, such as comb shape and size, are important in mating behavior in both sexes (Siegel and Dudley, 1963; Zuk et al., 1990; Pizzari et al., 2003). Males prefer to invest sperm in females with relatively large comb (Cornwallis and Birkhead, 2006); reciprocally, females preferentially like to copulate with dominant males with large combs (Graves et al., 1985; Zuk et al., 1990). Additionally, the comb is associated with body temperature regulation (van Kampen, 1971), egg production (Wright et al., 2008), and fecundity and sex maturity (Wright et al., 2012). From the above aspects, it can be concluded that the comb has potential economic value in poultry breeding.

Since the chicken genome was published (Wallis et al., 2004), researchers have made full use of this genomic data to identify the genetic differences under the phenotypic variation. Great progress has been made in understanding the genetic associations with some traits, such as yellow skin (Eriksson et al., 2008), silky-feather (Feng et al., 2014), and muffs and beard phenotypes (Guo et al., 2016). Also, numerous studies have explored the genetic mechanism for comb traits, for instance, the identification of variants and genes responsible for comb shape (Rose-comb and Pea-comb), comb mass (Johnsson et al., 2012), and comb color (Dong et al., 2019). However, traits related to comb size, such as comb length (CL) and comb height (CH), are less considered in genetic research.

The genome-wide association study (GWAS) is a powerful method for detecting causal variants of complex traits and has been widely used in livestock for the past decades. Many studies have successfully used GWAS for poultry breeding to identify quantitative trait loci (QTLs) for numerous desired traits, namely, bone size (Guo et al., 2020), internal organ traits (Moreira et al., 2019), and egg production (Liu et al., 2019). Because of the high cost of sequencing large samples, most of these studies were carried out using SNP chips. However, a limitation of SNP chips is that they can only detect pre-existing genetic variants. Compared with SNP chips, whole-genome sequencing (WGS) is more efficient and powerful in detecting and fine-mapping rare variants (Tam et al., 2019). With the decrease in sequencing costs, it is now more beneficial to explore genomic information using WGS.

In this study, to explore the potential genomic regions and candidate genes associated with size-related comb traits in Nandan-Yao chickens, we employed WGS to detect genome-wide genetic variation in a population of 300 Nandan-Yao chickens and performed a genome-wide association analysis on five comb traits.

## Materials and Methods

### Sample Collection and Phenotypic Measurement

The experimental animals used in this study were Nandan-Yao chickens. A total of 300 animals (149 males and 151 females) were randomly collected from Gangfeng Agriculture and Husbandry Co., Ltd., Guigang. All birds were housed in cages. The animals were slaughtered after 120 days, and the combs were cut off for phenotypic measurements. We collected five phenotypes in total, which were comb area (CA), comb thickness (CT), CL, CH, and comb weight (CW), respectively. CT was measured at the thickest point with a Vernier caliper, and CW was measured using a scale. For measurement of CA, CH, and CL, we delineated the contour of the comb on A4 paper along the comb’s edge and scanned the comb profile into the digital photo by the scanner; we then used the Photoshop Measurement feature to measure CA, CH, and CL. CH was measured from the point at which the comb met the head to the top of the highest spike, and CL was defined from end to end (Navara et al., 2012). To reduce the false positives caused by outliers in the association analysis, the records over triple standard deviation were removed. The phenotypic correlations among five comb traits were calculated using the Pearson method by “psych,” a package in R (version 3.6.2).

### Genomic Analysis

The genomic DNA was isolated from blood using the standard phenol–chloroform method, and the WGS data were generated in Novogene Bioinformatics Technology Co., Ltd., by Illumina PE150 platform. We selected the Gallus_gallus-5.0 (GCA_000002315.3) as the reference genome (Zerbino et al., 2018) and utilized BWA (version 0.7.8) for genome alignment (Li and Durbin, 2009) with parameters “mem -t 4 -k 32 -M.” Subsequently, the “mpileup” module from SAMtools (version 1.3.1) was used to detect genome-wide genetic variation, namely, single-nucleotide polymorphisms (SNPs) and small insertions/deletions (INDELs; Li et al., 2009). To obtain reliable SNPs, we further filtered the data with the following thresholds: the average depth per SNP >5, minor allele frequency >0.05, missing rate per individual <0.1, missing rate per SNP <0.1, and *p*-value of Hardy–Weinberg Equilibrium >10^–6^. The filtered process was performed using VCFtools (version 0.1.17) (Danecek et al., 2011) and PLINK (version 1.90) (Purcell et al., 2007). The remaining SNPs with missing genotypes were imputed using BIMBAM (version 1.0) software (Scheet and Stephens, 2006). Finally, all eligible SNPs derived from autosomes (GGA) were used for GWAS analysis. The filter parameters of INDELs were similar to those of SNPs but without imputation.

### Genome-Wide Association Studies

We performed a principal component analysis (PCA) using all autosomal SNPs before the association tests to estimate population stratification (Price et al., 2006). The top three PCs accounted for 2.28% of the total variance, suggesting that the differentiation of experimental chickens was not obvious. Besides, considering the effects of hormones on combs (Eitan et al., 1998; Symeon et al., 2012), we both fit the top three PCs and sex as covariates while executing association analysis. All association tests were carried out using GEMMA software (version 0.96) (Zhou and Stephens, 2012). The univariate mixed linear model was as follows:

y=W⁢α+x⁢β+u+e

where y is a vector of phenotypic values; W is a matrix of covariates (fixed effects) including sex and the first three PCs; α is a vector of the corresponding coefficients for fixed effects; x is a vector of genotypes; β is the effect size of the maker; u is a vector of random effects with a covariance structure as u ∼ N(0, G$\sigma_{\text{u}}^{2}$), where G is the genetic relatedness matrix calculated from all SNP markers and $\sigma_{\text{u}}^{2}$ is the polygenic additive variance; e is a vector of residual errors with e ∼ N(0, I$\sigma_{\text{e}}^{2}$), where I is the identity matrix and $\sigma_{\text{e}}^{2}$ is the residual variance.

For SNP-based GWAS, we selected the simpleM method (Gao et al., 2008; Gao, 2011) to adjust the threshold of the genome-wide significance *p*-value. After the simpleM test, a total of 6,971,926 effective independent tests were obtained. Hence, the significant and suggestive thresholds for SNP-based GWAS were set at 7.17 × 10^–9^ (0.05/6,971,926) and 1.43 × 10^–7^ (1/6,971,926), respectively. The SNPs that reached the suggestive genome-wide threshold would be annotated using ANNOVAR software (Wang et al., 2010).

We also carried out the INDEL-based GWAS for comb traits using the same analytical process as that used for SNP-based GWAS. The significant and suggestive thresholds for INDEL-based GWAS were set at 7.31 × 10^–8^ (0.05/684,401) and 1.46 × 10^–6^ (1/684,401), respectively, where 684,401 is the number of INDELs we used for GWAS. Finally, we used the “vt” tool to make INDELs normalized (Tan et al., 2015) for annotation.

The Manhattan and quantile--quantile plots for GWAS results were performed using the ‘‘CMplot’’ package,^1^ and the diagram for regional plot on GGA 6 was created by “karyoploteR” package (Gel and Serra, 2017) in the R (version 3.6.2). The linkage disequilibrium (LD) correlation (*r*^2^) between the associated SNPs (genome-wide suggestive and significant loci on GGA 6) was estimated using PLINK and visualized by LDBlockShow release 1.35.^2^ The conditional GWAS for lead SNP was performed by GCTA software (version 1.26) (Yang et al., 2011).

## Results

### Phenotypic Statistics

Descriptive statistics of the five comb traits are presented in Table 1, namely, minimum, maximum, mean, standard deviation, and coefficient of variation (CV). We observed that CW had the highest CV in both sexes (36.90% for males and 48.96% for females), followed by CA (29.58% for males and 41.35% for females). The phenotypic correlations for the five comb traits ranged from 0.93 to 0.97, while CW and CA had the strongest correlation (Supplementary Figure 1).

**TABLE 1:** Descriptive statistics for comb traits.

**Trait^a^**	**Number^b^**	**Males**					**Females**				
		**Min**	**Max**	**Mean**	**SD**	**CV (%)**	**Min**	**Max**	**Mean**	**SD**	**CV (%)**
CT (mm)	300	10.20	24.23	17.42	2.80	16.06	3.14	12.05	6.04	1.85	30.63
CW (g)	298	4.26	32.40	15.48	5.71	36.90	0.48	6.92	2.20	1.10	49.86
CA (cm^2^)	286	10.47	58.55	32.20	9.52	29.58	1.01	17.42	6.98	2.89	41.35
CL (cm)	280	5.55	12.40	9.09	1.21	13.31	2.69	7.47	4.75	0.84	17.63
CH (cm)	280	2.67	7.07	5.05	0.82	16.27	0.90	3.50	2.28	0.52	22.78

*^*a*^CT, comb thickness; CW, comb weight; CA, comb area; CL, comb length; CH, comb height.*

*^*b*^The remaining measurements after removing over triple standard deviation.*

### Summary of Sequencing and Population Stratification

We obtained approximately 2,999 Gb of clean data from 300 Nandan-Yao chickens. The average alignment rate and depth were 98.75% and 8.38×, respectively. The coverage of at least 1× per sample was 82.7–93.49%. Detailed information on sequencing is shown in Supplementary Table 1, and the PCA plot is presented in Supplementary Figure 1.

### SNP-Based GWAS

**FIGURE 1:**
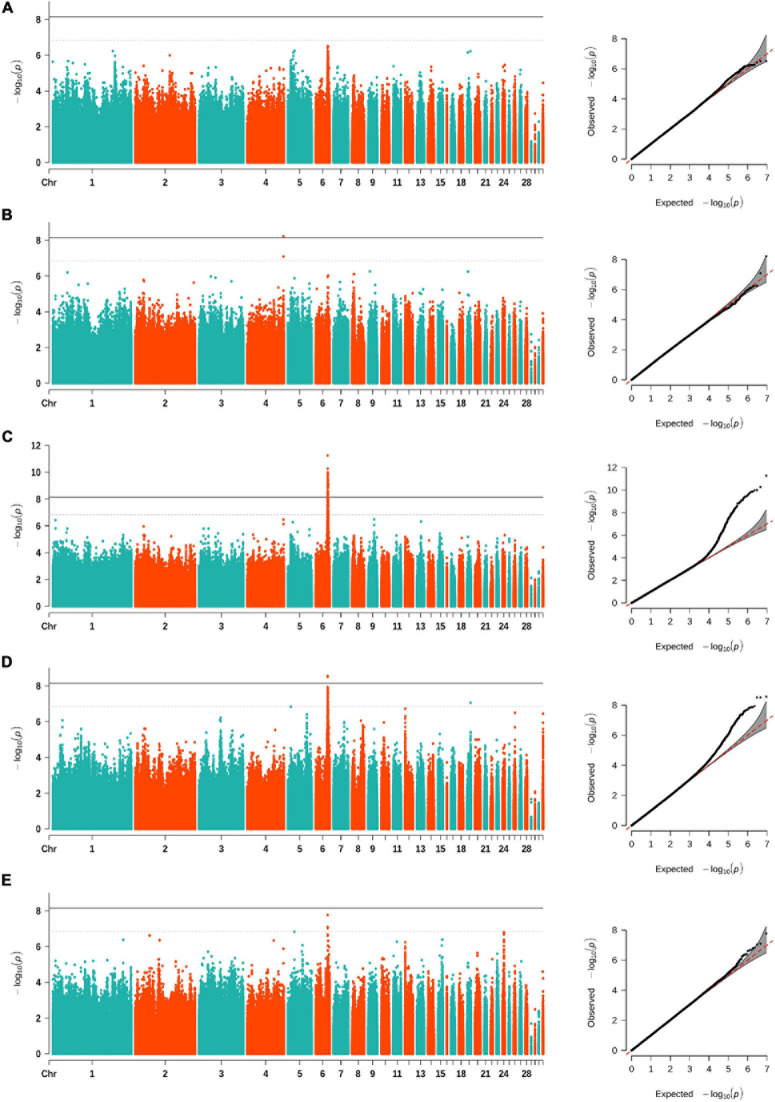
Manhattan (left) and quantile–quantile (Q–Q) (right) plots of SNP-based GWAS for comb area **(A)**, comb height **(B)**, comb length **(C)**, comb thickness **(D)**, and comb weight **(E)**. The Manhattan plots show −log_10_(observed *p*) for SNPs (y-axis) against their corresponding positions on each chromosome (x-axis), while the Q–Q plots show the observed and expected −log_10_(*p*). The horizontal dashed and solid lines in Manhattan plots represent the genome-wide suggestive (−log_10_(*p*) = 6.84) and significant (−log_10_(*p*) = 8.14) thresholds, respectively.

After filtering the SNPs, a total of 9,080,580 autosomal SNPs and 299 Nandan-Yao chickens were left for SNP-based GWAS. The distribution of all eligible SNPs on GGA 1–33 is shown in Supplementary Figure 1. The univariate GWAS was performed on CT, CW, CA, CL, and CH, respectively. The results showed that a prominent genomic region spanning from 29.6 to 31.4 Mb on GGA 6 was associated with three comb traits, namely, CT, CW, and CL (Figure 1). Precisely, in this region, we identified 132 SNPs suggestively associated with CL (including 47 significant SNPs), 39 SNPs suggestively associated with CT (including 3 significant SNPs), and 3 SNPs suggestively associated with CW, respectively (Table 2). The SNPs that reached the suggestive threshold for CL contained all associated SNPs identified for CT and CW (Supplementary Figure 2). Besides, an SNP located on GGA 4 (Figure 1) was found to be significantly associated with CH (−log_10_(*p*) = 8.22). Unfortunately, no SNP reached the suggestive threshold for CA.

**TABLE 2:** A descriptive summary of associated SNPs on GGA 6.

**Chromosome**	**Position**	**N_Sugg^*a*^**	**N_Sig^*b*^**	**Lead variant**	**−log_10_(*p*)^*c*^**	**Beta estimates^*d*^**	**Trait^*e*^**
6	30,237,969–30,946,600	39	3	6:30,354,876	8.56	−1.96	CT
6	30,352,191–30,354,876	3	0	6:30,354,876	7.77	−3.25	CW
6	29,620,815–31,440,202	132	47	6:30,264,318	11.26	−1.22	CL

*^*a*^The number of SNPs with −log_10_(*p*) > 6.84.*

*^*b*^The number of significant SNPs with −log_10_(*p*) > 8.14.*

*^*c*^The −log_10_(*p*) of lead variant.*

*^*d*^The beta estimates of lead SNP.*

*^*e*^CT, comb thickness; CW, comb weight; CL, comb length.*

As shown in Table 2, the same lead SNP located at 30,354,876 bp on GGA 6 was observed in CW and CT. Meanwhile, the lead SNP for CL was located at 30,264,318 bp on GGA 6. These two loci were both located in the intergenic region between the *MIR7472* and *MCMBP* genes. Interestingly, the two SNPs had a low LD correlation (*r*^2^ = 0.3817). For CH, the most significantly associated SNP (4:89,933,310) was located within an intron of the *GFRA4* gene. The detailed information of all associated SNPs is provided in Supplementary Table 2.

### INDEL-Based GWAS

**FIGURE 2:**
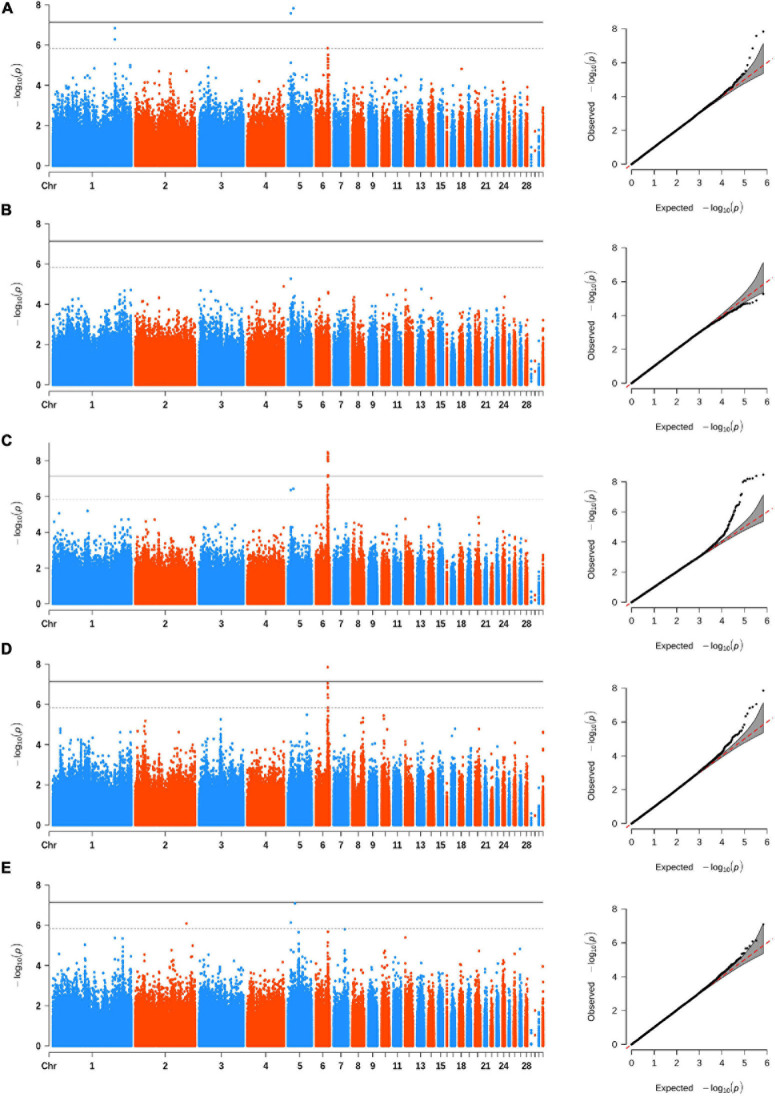
Manhattan (left) and quantile–quantile (Q–Q) (right) plots of INDEL-based GWAS for comb area **(A)**, comb height **(B)**, comb length **(C)**, comb thickness **(D)**, and comb weight **(E)**. The Manhattan plots indicate −log_10_(observed *p*) for INDELs (y-axis) against their corresponding positions on each chromosome (x-axis), while the Q–Q plots show the observed and expected −log_10_(*p*). The horizontal dashed and solid lines in Manhattan plots represent the genome-wide suggestive (−log_10_(*p*) = 5.84) and significant (−log_10_(*p*) = 7.14) thresholds, respectively.

We carried out the INDEL-based GWAS for five comb traits with 684,401 INDELs and identified 25 associated INDELs (Table 3). Interestingly, we found that the same region on GGA 6 was strongly associated with CL, CA, and CT (Figure 2), which implicated 19 INDELs (Supplementary Figure 2). The most significantly associated variant was a 2-bp deletion, located from 30,376,404 to 30,376,405 bp in the intergenic region between the *MIR7472* and *MCMBP* genes. We also annotated several genes from the associated INDELs on GGA 1, 2, and 5, namely, *ACOD1*, *UCHL3*, *CPQ*, *CYB5R2*, *IGF2*, *MRPL23*, and *FGF3* (Table 3).

**TABLE 3:** Genes linked to the 25 associated INDELs for comb traits in Nandan-Yao chickens.

**Chromosome**	**Position^*a*^ (bp)**	**Ref^*b*^**	**Alt^*c*^**	**Trait^*d*^**	**−log_10_(*p*)**	**Annotation**	**Nearby gene**
1	154,683,824	–	A	CA	6.28	Intergenic	*ACOD1*; *UCHL3*
1	154,805,766	–	CAAGGAGG	CA	6.84	Intergenic	*ACOD1*; *UCHL3*
2	127,710,350	–	C	CW	6.07	Intergenic	*CPQ*; *RPL30*
5	7,403,641–7,403,642	TC	–	CW, CL, CA	6.13–7.58	Intronic	*CYB5R2*
5	13,977,826	A	–	CL, CA	6.44–7.83	Intergenic	*IGF2*; *MRPL23*
5	17,679,480	–	T	CW	7.09	Intergenic	*FGF3*; *CTTN*
6	29,621,960–29,621,961	CA	–	CL	5.99	Intergenic	*VAX1*; *PRLHR*
6	29,923,004–29,923,010	CCCCCTC	–	CL	5.84	Intergenic	*CACUL1*; *FAM45A*
6	29,944,104	–	A	CL	6.08	Intergenic	*CACUL1*; *FAM45A*
6	29,948,947	–	AT	CL	6.65	Intergenic	*CACUL1*; *FAM45A*
6	29,961,899–29,961,902	AAGG	–	CL	6.28	Intergenic	*CACUL1*; *FAM45A*
6	30,237,444	A	–	CT, CL	6.49–8.07	Intergenic	*MIR7472*; *MCMBP*
6	30,271,529	C	–	CA, CT, CL	5.84–8.20	Intergenic	*MIR7472*; *MCMBP*
6	30,310,013	–	G	CT, CL	6.88–8.19	Intergenic	*MIR7472*; *MCMBP*
6	30,376,404–30,376,405	TA	–	CT, CL	7.85–8.48	Intergenic	*MIR7472*; *MCMBP*
6	30,571,297	–	T	CT, CL	6.81–7.11	Intergenic	*MCMBP*; *FGFR2*
6	30,878,266	T	–	CL	6.1	Intergenic	*MCMBP*; *FGFR2*
6	30,889,738	C	–	CL	8.07	Intergenic	*MCMBP*; *FGFR2*
6	30,911,206–30,911,207	AT	–	CT, CL	6.33–8.23	Intronic	*FGFR2*
6	30,911307	–	CATT	CL	7.98	Intronic	*FGFR2*
6	30,943,119	–	TGTG	CL	6.41	Intronic	*FGFR2*
6	31,073,199–31,073,201	TTG	–	CL	8.39	Intronic	*ATE1*
6	31,083,785	G	–	CL	6.46	Intronic	*ATE1*
6	31,088,999	–	G	CL	7.18	Intronic	*ATE1*
6	31,440,157	–	C	CL	6.50	Intergenic	*ATE1*; *IKZF5*

*^*a*^The normalized position of INDELs.*

*^*b,c*^Insertion or deletion.*

*^*d*^CT, comb thickness; CW, comb weight; CA, comb area; CL, comb length; CH, comb height.*

### Analysis of LD and Conditional GWAS

**FIGURE 3:**
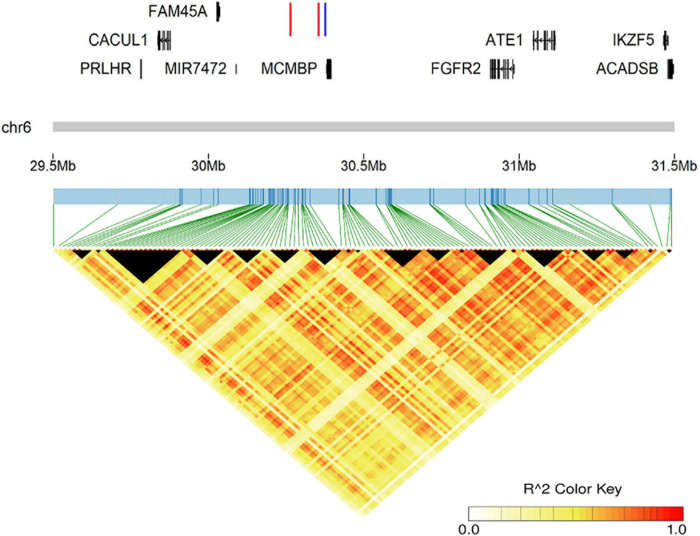
The genomic information of the prominent region on GGA 6 and LD analysis for all suggestively associated SNPs. The two red lines represent two lead SNPs (6:30,354,876 for comb weight and comb thickness, 6:30,264,318 for comb length), while the blue line represents the lead INDELs (6:30,376,404–30,376,405 for comb length and comb thickness).

We performed LD analysis on prominent signals for SNP-based GWAS; the LD correlation for all suggestive loci on GGA 6 from 29.6 to 31.4 Mb is shown in Figure 3. Unexpectedly, we found that the two lead SNPs (6:30,354,876 for CT and CW, 6:30,264,318 for CL) had a low level of LD correlation (*r*^2^ = 0.3817). Therefore, to test if the independently associated SNPs existed in the signal region for relevant comb traits, we subsequently carried out an approximately conditional GWAS based on the lead SNPs for CT, CW and CL. As the results showed, the level of significant and suggestive loci around the lead SNPs was all decreased below the suggestive genome-wide threshold (−log_10_(*p*) = 6.84) after conditional analysis (Supplementary Figure 3), indicating that no multiple independently associated variants existed in the signal region on GGA 6.

## Discussion

To date, relatively few GWAS have been performed on comb traits, among which only two studies are established on the quantitative characters of combs. One was conducted on CL, CH, and CW using 600 K SNP arrays (Shen et al., 2016) and the other on CA using a 60 K SNP chip (Lien et al., 2017). Compared with these two studies, the magnitude of SNPs used in our study was much larger. Furthermore, our association tests were conducted on genome-wide INDELs, which are rarely used in GWAS research for chickens. However, a limitation is that our sample size was small for GWAS, which led us to remove variants with low frequency. Further GWAS with a larger sample size might result in the identification of additional variants in the future. Overall, the WGS data generated in this study can be used in conjunction with subsequent studies to provide more useful information.

### Benefits for Local Poultry Industries

The Nandan-Yao chicken is one of the most popular indigenous breeds in the Guangxi Zhuang Autonomous Region. However, local poultry industries mostly breed Nandan-Yao chickens based on phenotypes, resulting in slow genetic progress. The appropriate use of molecular marker-assisted breeding can accelerate the selection of Nandan-Yao chickens to obtain the desired phenotypes, allowing poultry companies to increase profits more quickly. In our study, we identified 134 SNPs and 25 INDELs that were strongly associated with comb size, which could be helpful in the selection of Nandan-Yao chickens. For example, the lead SNP (6:30,354,876, rs737686019) identified from CT and CW could be considered a priority for selection. From the SNP-based GWAS, we could find that the C allele of rs737686019 had negative effects on CW and CT (Table 2). Therefore, by removing animals with the C allele, the comb size of the population might increase modestly. This would be much faster than screening based on the phenotypes directly. In addition, due to the associated SNPs on GGA 6 are highly correlated, breeders could choose other SNPs as candidate markers for comb traits by selecting the loci with more obvious effects.

### Candidate Genes Associated With Comb Traits

Two genes were considered as candidate genes for comb development, one is *FGFR2* (fibroblast growth factor receptor 2) and the other is *CYB5R2* (cytochrome b5 reductase 2). For *FGFR2*, there were several reasons: (1) we identified this gene from both SNP- and INDEL-based GWAS, suggesting that it was highly associated with comb traits; (2) it is known that the comb comprises three layers, namely, epidermis, dermis, and central connective tissue (Nakano et al., 1996); meanwhile, a previous study reported that *FGFR2* can be used as a gene marker for epithelial cell migration and proliferation (Carre et al., 2018); therefore, we speculated that *FGFR2* might be involved in the structural formation of the comb; (3) some studies have reported that, in human, a mutation in the 2c splice variant of the *FGFR2* gene results in Crouzon syndrome (Reardon et al., 1994), as well as regulates coronal suture development (Pfaff et al., 2016); additionally, in chicken, a previous study (Wilke et al., 1997) showed that *FGFR2* transcripts were expressed throughout the head mesenchyme before budding out of facial prominences (from Hamburger–Hamilton stage 9 to 17 of chick embryo) and expressed throughout the frontonasal mass during early facial prominence formation (Hamburger–Hamilton stage 20); collectively, these studies indicate that *FGFR2* is critical for facial or craniofacial development, which could further indicate an indirect influence on comb development. Indeed, the receptor protein encoded by *FGFR2* is a receptor tyrosine kinase (RTK), suggesting that *FGFR2* may be involved in comb development through the RTK/Ras/MAPK signaling pathway. However, this inference needs to be supported by additional evidence. For *CYB5R2* gene, we identified it from a deletion (5:7,403,641–7,403,642) in INDEL-based GWAS. It is a member of flavoprotein pyridine nucleotide cytochrome reductase family and is involved in many physiological processes (O’Leary et al., 2016). Collagen is known to be one of the major chemical compositions of comb (Nakano et al., 1996); meanwhile, a previous study reported that the expression of *CYB5R2* was significantly correlated with collagen maturation (Liu et al., 2015). Thus, we speculate that *CYB5R2* has a potential role in comb development. In addition, the region that contains *CYB5R2* on GGA 5 has been reported to be associated with head width (Gao et al., 2011), carcass weight, and body weight (Wang et al., 2012), implying that this genomic region is closely associated with chicken growth. However, because we selected these two genes as candidate genes primarily based on our inferences from previous research, more experiments and evidence are needed to verify their function in comb development.

## Conclusion

Using GWAS for comb traits with autosomal SNPs and INDELs derived from the WGS, the current study identified 134 SNPs and 25 INDELs strongly associated with comb traits, and two genes, *FGFR2* and *CYR5B2*, could be considered candidate genes for comb traits. The markers identified in our study could provide genomic targets for genetic improvement of comb traits in Nandan-Yao chickens. Additionally, continued investigation of these two candidate genes will help researchers further understand the genetic mechanism and identify causal variants underlying comb development.

## Data Availability Statement

The SNP and INDEL data generated from this study can be found in the European Bioinformatics Institute with the accession number PRJEB46210.

## Ethics Statement

The animal study was reviewed and approved by Guangxi University’s Animal Care and Use Committee.

## Author Contributions

ZY performed the GWAS and wrote the manuscript. LZo performed the statistical analysis of the phenotype. JD, YJ, and JJ contributed to the collection of samples and data. TS, WX, and LZe extracted the genomic DNA. XY contributed to the design of the study and the review of the manuscript. All authors read and approved the final manuscript.

## Conflict of Interest

The authors declare that the research was conducted in the absence of any commercial or financial relationships that could be construed as a potential conflict of interest.

## Publisher’s Note

All claims expressed in this article are solely those of the authors and do not necessarily represent those of their affiliated organizations, or those of the publisher, the editors and the reviewers. Any product that may be evaluated in this article, or claim that may be made by its manufacturer, is not guaranteed or endorsed by the publisher.

## Footnotes

1
https://github.com/YinLiLin/CMplot

2
https://github.com/BGI-shenzhen/LDBlockShow

## Supplementary Material

Supplementary Figure 1 **(A)** Pearson correlation coefficients for five comb traits. **(B)** The principal component plot of Nandan-Yao chickens. **(C)** The SNP distribution in Nandan-Yao chicken, the different colors indicate the number of SNPs within 0.5 Mb window size. **(D)** The INDEL distribution in Nandan-Yao chicken, the different colors indicate the number of INDELs within 1 Mb window size.

Supplementary Figure 2 Venn diagrams of associated SNPs and INDELs identified on GGA 6 for comb traits.

Supplementary Figure 3 Conditional GWAS in the prominent region on GGA 6. Regional association results of before **(left)** and after **(right)** conditional GWAS for CL, CT, and CW. The red dots represent the two lead SNPs for each trait (6:30,264,318 for CL, 6:30,354,876 for CT and CW). The blue dots indicate the −log_10_(*P*) of SNPs greater than 6.84 (suggestive genome-wide threshold) before conditional GWAS.

Supplementary Table 1 Detailed information about the whole-genome sequencing results.

Supplementary Table 2 The detailed information of all suggestively associated SNPs. The text in bold represents the lead SNP in each trait.
