# Supplementary figures and images for: Genome-Wide Association Study Using Whole-Genome Sequencing Identifies a Genomic Region on Chromosome 6 Associated With Comb Traits in Nandan-Yao Chicken
Source: Front Genet. 2021 Aug 2;12:682501. doi: 10.3389/fgene.2021.682501 (PMC8365347; doi:10.3389/fgene.2021.682501)

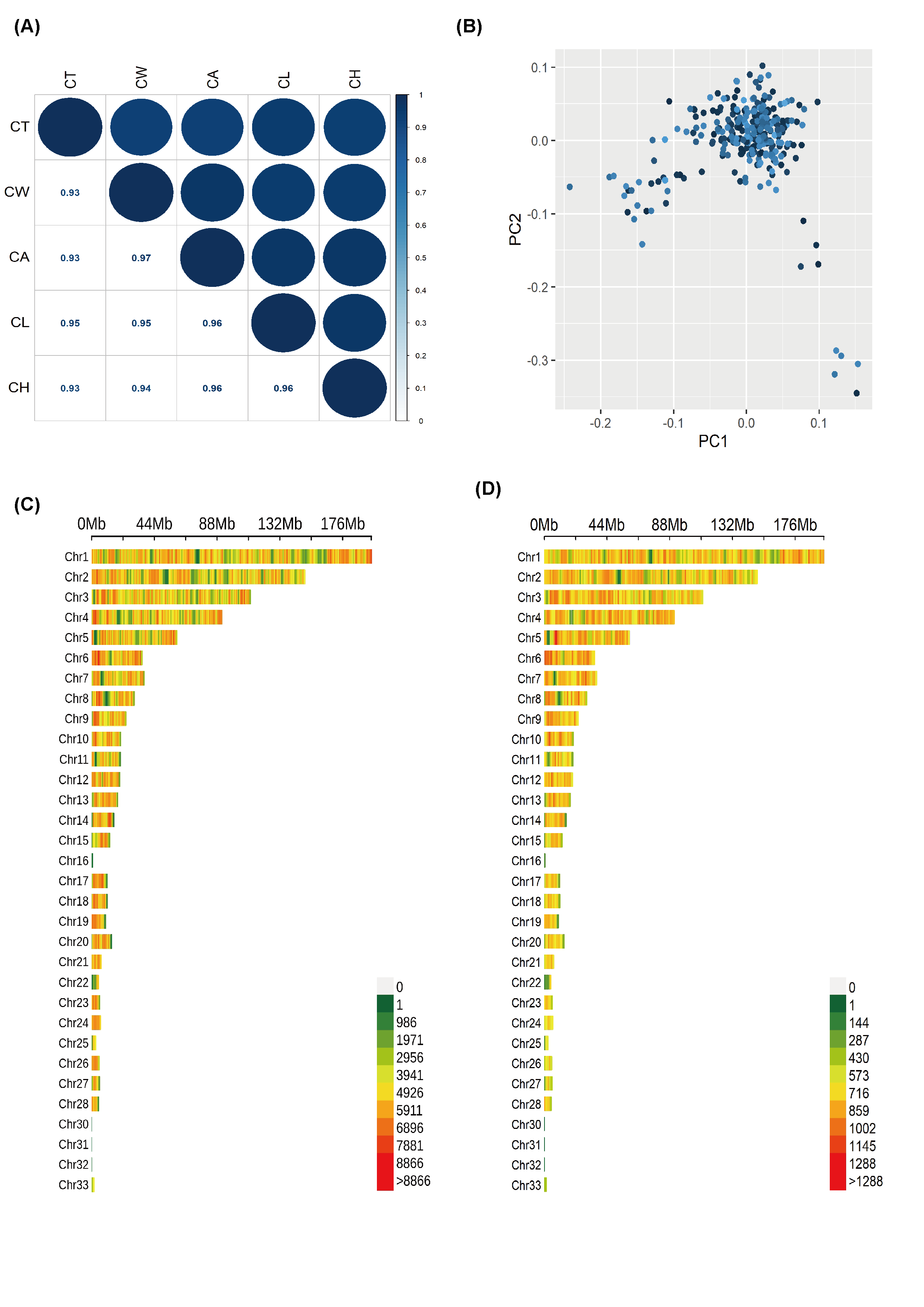

Supplement: Supplementary Figure 1 — (A) Pearson correlation coefficients for five comb traits. (B) The principal component plot of Nandan-Yao chickens. (C) The SNP distribution in Nandan-Yao chicken, the different colors indicate the number of SNPs within 0.5 Mb window size. (D) The INDEL distribution in Nandan-Yao chicken, the different colors indicate the number of INDELs within 1 Mb window size. [file Image_1.TIF]

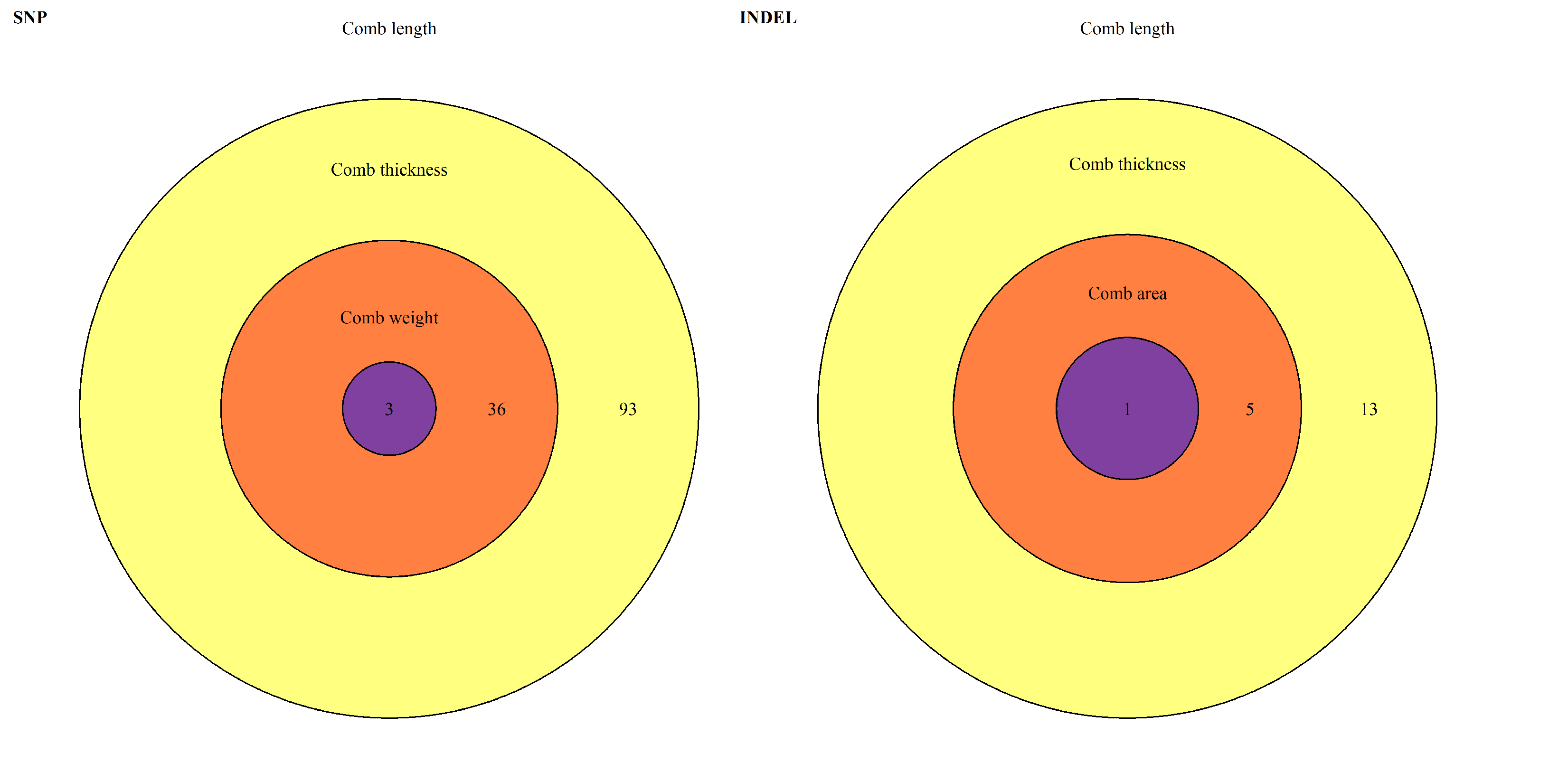

Supplement: Supplementary Figure 2 — Venn diagrams of associated SNPs and INDELs identified on GGA 6 for comb traits. [file Image_2.TIF]

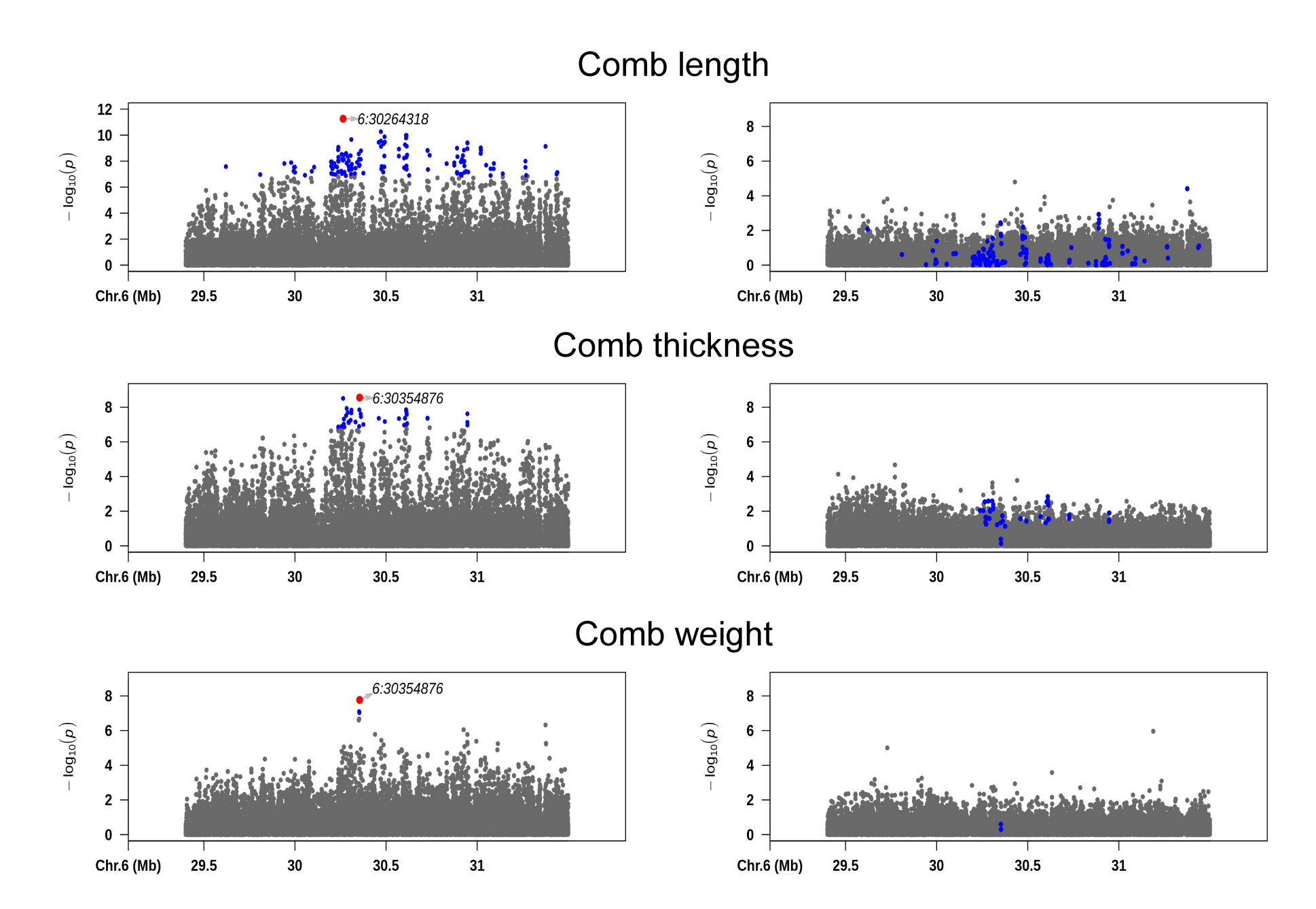

Supplement: Supplementary Figure 3 — Conditional GWAS in the prominent region on GGA 6. Regional association results of before (left) and after (right) conditional GWAS for CL, CT, and CW. The red dots represent the two lead SNPs for each trait (6:30,264,318 for CL, 6:30,354,876 for CT and CW). The blue dots indicate the −log10(P) of SNPs greater than 6.84 (suggestive genome-wide threshold) before conditional GWAS. [file Image_3.TIF]
